# Geoinformation-based landslide susceptibility mapping in subtropical area

**DOI:** 10.1038/s41598-021-03743-5

**Published:** 2021-12-21

**Authors:** Xiaoting Zhou, Weicheng Wu, Yaozu Qin, Xiao Fu

**Affiliations:** Key Laboratory of Digital Lands and Resources and Faculty of Earth Sciences, East China University of Technology, Nanchang, 330013 Jiangxi China

**Keywords:** Environmental sciences, Natural hazards

## Abstract

Mapping susceptibility of landslide disaster is essential in subtropical area, where abundant rainfall may trigger landslide and mudflow, causing damages to human society. The purpose of this paper is to propose an integrated methodology to achieve such a mapping work with improved prediction results using hybrid modeling taking Chongren, Jiangxi as an example. The methodology is composed of the optimal discretization of the continuous geo-environmental factors based on entropy, weight of evidence (WoE) calculation and application of the known machine learning (ML) models, e.g., Random Forest (RF), Support Vector Machine (SVM) and Logistic Regression (LR). The results show the effectiveness of the proposed hybrid modeling for landslide hazard mapping in which the prediction accuracy vs the validation set reach 82.35–91.02% with an AUC [area under the receiver operating characteristic (ROC) curve] of 0.912–0.970. The RF algorithm performs best among the observed three ML algorithms and WoE-based RF modeling will be recommended for the similar landslide risk prediction elsewhere. We believe that our research can provide an operational reference for predicting the landslide hazard in the subtropical area and serve for disaster reduction and prevention action of the local governments.

## Introduction

Landslide is a common geological disaster leading to destruction and damages to human society in subtropical areas. With the socioeconomic development and the continuous expansion of human activities into the natural environment, landslide occurs more and more frequently and constitutes the main disaster threatening the safety of life and restricts the economic development in the hilly and mountainous areas^1–4^. Accurate and reliable mapping of landslide risk is a key step for local decision-makers and authorities to plan reasonable land use and implement disaster reduction and prevention measures to reduce the massive damage^5–9^.

Actually, a number of scientists have been exploring reliable approaches for landslide hazard mapping^10,11^. With the advent of geoinformation technology including remote sensing (RS), Geographic Information System (GIS), Global Positioning System (GPS) or Beidou System (BDS) and powerful computer processing facility, acquisition and processing of geo-environmental factors with high resolution have been greatly facilitated^8,12^. The prediction of landslide hazard has been also upgraded from knowledge-driven qualitative analysis to data-driven quantitative modeling^13–15^. The knowledge-driven model is to sort out and weight the limited landslide influencing factors based on a priori knowledge to conduct a landslide susceptibility mapping^16,17^, while the data-driven modeling is to achieve the same purpose but able to avoid the subjective uncertainty of experts and has higher accuracy and reliability^17–20^.

Statistical analysis and machine learning (ML) modeling are two major data-driven approaches. The calculation process of the statistical models such as frequency ratio (FR), certainty coefficient (CF), information value (IV) and weight of evidence (WoE) is simple; and qualitative or categorical factors can be converted into quantitative weights by these approaches, and thence, they are widely employed for landslide risk assessment^15,21–23^. However, the statistical models are sensitive to the nonlinear phenomena which require specific algorithms to sort them out^23,24^.

Since the appearance of artificial intelligence, different ML algorithms including deep learning have been applied in the field of landslide risk mapping^11,25–28^. Based on the target definition, or rather, collection of samples for training, ML approaches can automatically analyze and extract rules from the input data to make predictions^14^. Meanwhile, it is highly efficient in calculating high-dimension data and can fit the nonlinear relationships between target and factors^8,29–31^. Nevertheless, the prediction accuracy of the most studies, even including those harnessing the hotspotted deep learning techniques^32–35^, comes between 75 and 85%, except for those of Huangfu et al.^36^, Ou et al.^26^, Zhang et al.^27^ and Zhou et al.^28^, who have achieved landslide risk prediction with an accuracy of 86–94.54%. This is not ideal for government to target effectively and accurately the high risk zones for implementing disaster reduction and prevention measures in the subtropical areas. Hence, it is necessary to effectuate some improvement in certain technical aspect of the ML approaches.

It has been decades since hybrid models were proposed for landslide risk assessment. Hybrid models are in fact constructed by integrating two or more models in aspect of sample selection^28,37^, feature selection^21,38^, information extraction and finally landslide hazard prediction with reasonable accuracy^10,22,25,39–41^. Hence, hybrid modeling has gained recently a momentum in improving the accuracy and reliability of landslide risk mapping^26,36,40,42,43^. However, there are still uncertainty in processing both categorical and continuous factors which may influence directly the prediction accuracy.

Based on the above understanding, the main objective of this study is to improve the landslide risk modeling and prediction using hybrid models by coupling WoE with ML algorithms such as Logistic Regression (LR), Support Vector Machine (SVM) and Random Forest (RF) taking Chongren, Jiangxi, China, a typical county in the subtropical area, as an example. A specific objective is to test the effectiveness of the discretization approach based on entropy to see whether it can bring us the expected improvement while discretizing the continuous factors.

## Data and methodology

**Figure 1:**
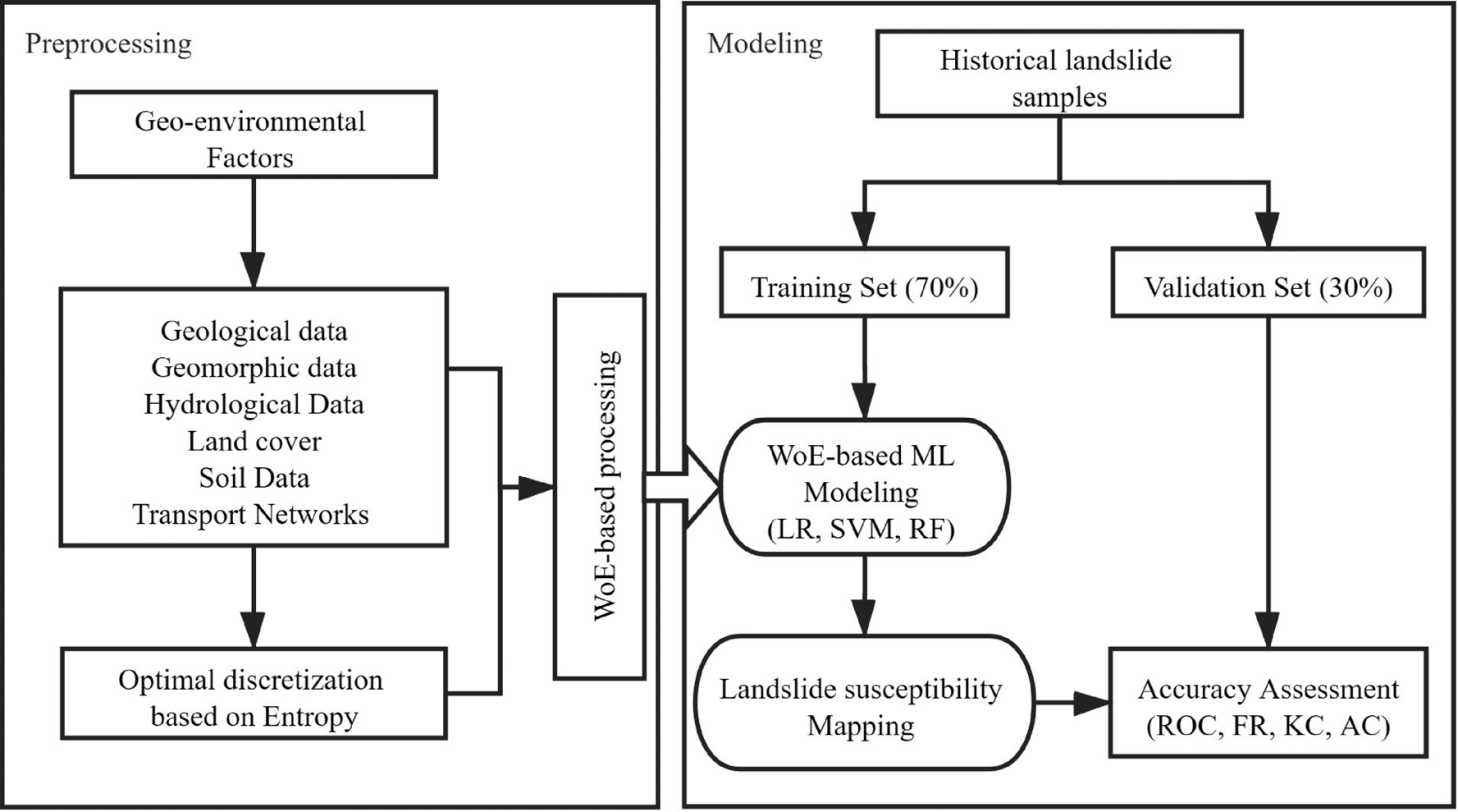
Methodological flowchart.

The methodological procedures involved in the research are depicted as follows: (1) data preparation of landslide samples and geo-environmental factors; (2) entropy-based optimal discretization of the continuous factors; (3) WoE-based processing of both continuous and categorical geo-environmental factors and establishment of the hybrid models; (4) modeling and mapping of landslide susceptibility; (5) accuracy assessment and validation of the proposed models (Fig. 1).

### Study area

**Figure 2:**
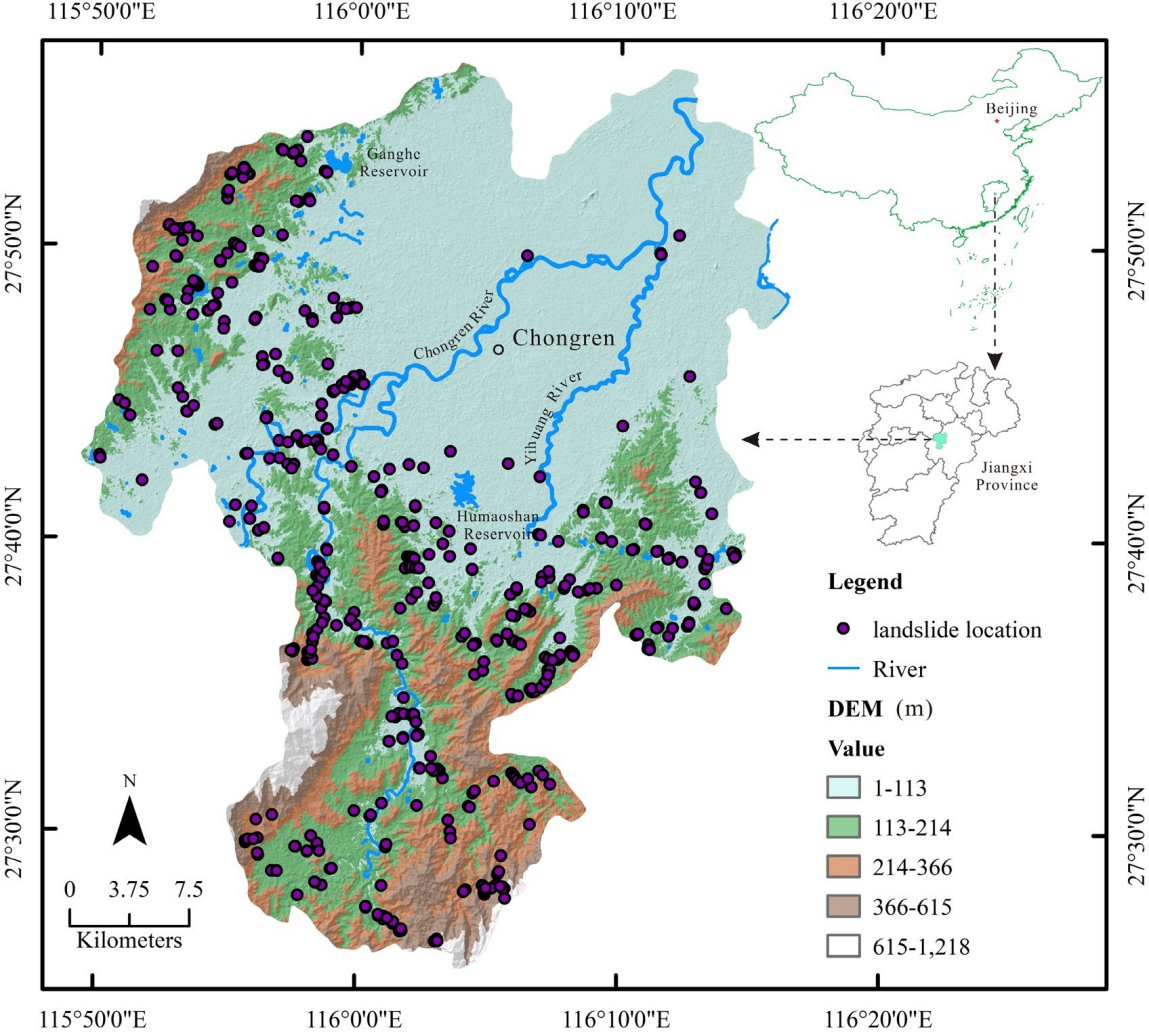
Geographical location of Chongren and distribution of the historical landslides. The map was created using ArcGIS version 10.6 (https://www.esri.com/).

Chongren is a county situated in the central part of Jiangxi, within the extent of longitude from 115° 49′ 16″ E to 116° 16′ 55″ E and latitude from 27° 24′ 29″ N to 27° 57′ 29″ N (Fig. 2), encompassing an area of 1520 km^2^. The general landform is an incomplete hilly basin surrounded by mountains on three sides and opening toward the northeast. The annual average temperature from 1981 to 2010 is 17.6 °C, and the annual average precipitation from 1959 to 2017 is 1783.8 mm driven by monsoon in the subtropical climate zone. There are more than 140 small rivers or streams in the study area with an accumulated running course of 910 km. All these rivers or streams constitute a part of the Fuhe River watershed as tributaries and subtributaries. Geologically, the exposed strata are from the Upper Proterozoic, e.g., Sinian (Nanhua) to the Upper Palaeozoic, e.g., Devonian, Carboniferous, and to the Mesozoic, i.e., Triassic, Jurassic, and Cretaceous and at last the Quaternary. Since the Proterozoic era, the study area had experienced sedimentation, magmatism, tectonism and metamorphism with intense and complex development and transformation, forming a complex structural pattern composed of tectonic entities such as ductile faults, superimposed folds, brittle faults and depression basins.

Regarding the geological disasters, small-scale shallow landslides are dominant in the study area. After slope cutting for infrastructure construction, the natural loose deposits (i.e., soil) or cracked rock masses (mainly phyllitic slate and rocks with downslope bedding or fracture) lose support and balance, forming a new free dangling surface. In case of heavy rainfall, the slope slips downward due to heavy load and instability. Such landslides generally have no signs, and the time from creeping to occurrence of an obvious slip is short, which, therefore, often causes major geological disasters leading to house collapse and casualties. Moreover, in the site of such landslides, a new scarp (or back wall) is formed, inducing the generation of new landslides at the trailing edge of the slope. This process is the same as the development of headward erosion in a slope valley, producing a chain of landslides.

Field investigation revealed that heavy rains triggered several landslides near the town Xiangshan on July 7, 2019, severely blocking the traffic with more than 30,000 m^3^ of landslide bodies; and on August 23, 2017, a landslide with a total volume of about 10,000 m^3^ occurred in the village Pingshan due to a rainstorm, causing power outage, interruption of telecommunication and severe road congestion.

### Field observation data

**Figure 3:**
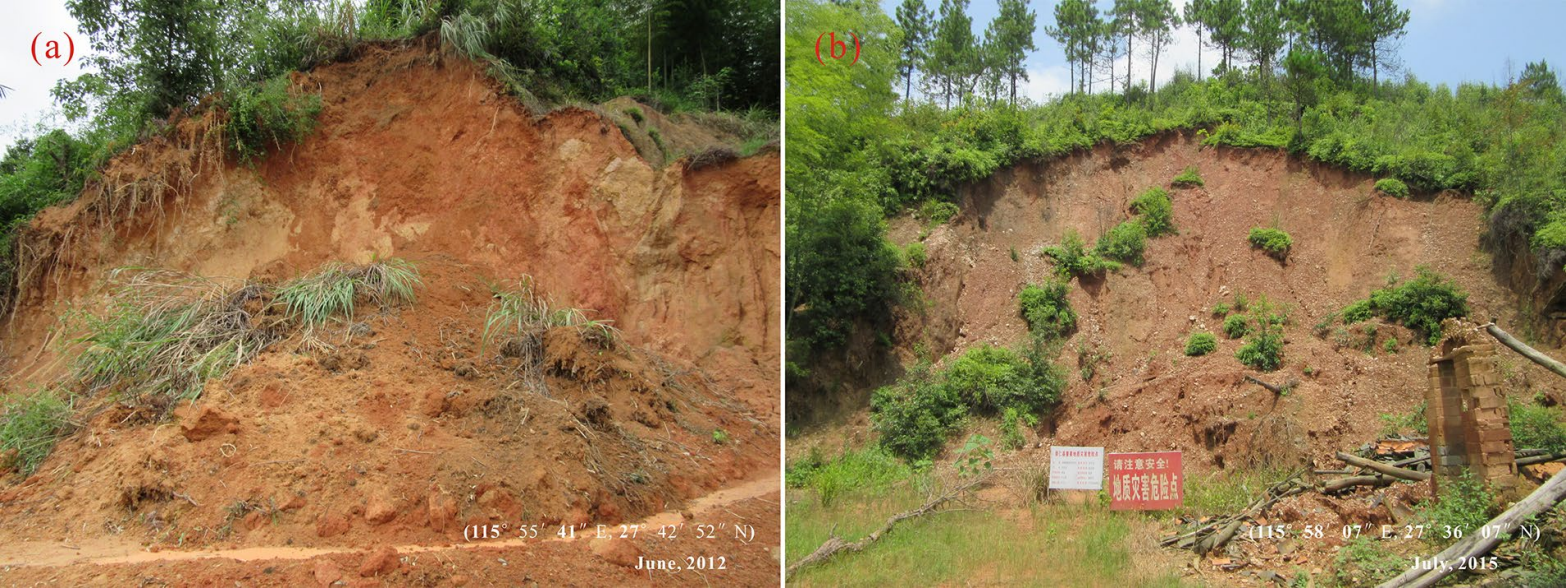
Photos of the rainfall triggered landslides in the study area.

The prediction of landslide disaster based on data-driven method is to calculate the probability of landslide occurrence in the study area by fitting the relationship between the historical landslides and the geo-environmental factors^44^. A detailed field survey of the historical landslides in the past decade was conducted in Chongren during the campaign of 1/50,000 Geological Disaster Survey by the 264 Geological Brigade of Jiangxi Nuclear Industry in 2017 and 588 landslides that took place in the period 2008–2017 (Fig. 3) were obtained as points. In reference to Google Earth (©Google) images, these landslide points were verified and vectorized into polygons. Meanwhile, the same number of stable points were stochastically selected in the stable areas, e.g., where the slope is less than 3°. A value of 1 was assigned to landslides and 0 to non-landslide points. As proposed by Zhang et al.^27^, Huangfu et al.^36^, Ou et al.^26^, and Zhou et al.^28^, 70% of the landslides and non-landslide samples were randomly picked out to constitute a training set (TS) to model landslide susceptibility, and the remained landslides and non-landslide samples (30%) as a validation set (VS) to evaluate the accuracy of modeling.

### Geo-environmental factors

#### Preparation

The occurrence of landslides is a consequence of the long-term joint action of the endogenous factors, i.e., geology, landform, vegetation and soil, etc., and the short-term predisposing factors, i.e., rainfall, earthquake and anthopogenic activities^18,27^. According to previous research on the landslide-causative factors^27,28,36^ and landslide field investigation in Chongren, geological and geomorphological data, hydrological data, land cover and transport system data were used to establish geoinformation datasets for landslide hazard analysis.

**Figure 4:**
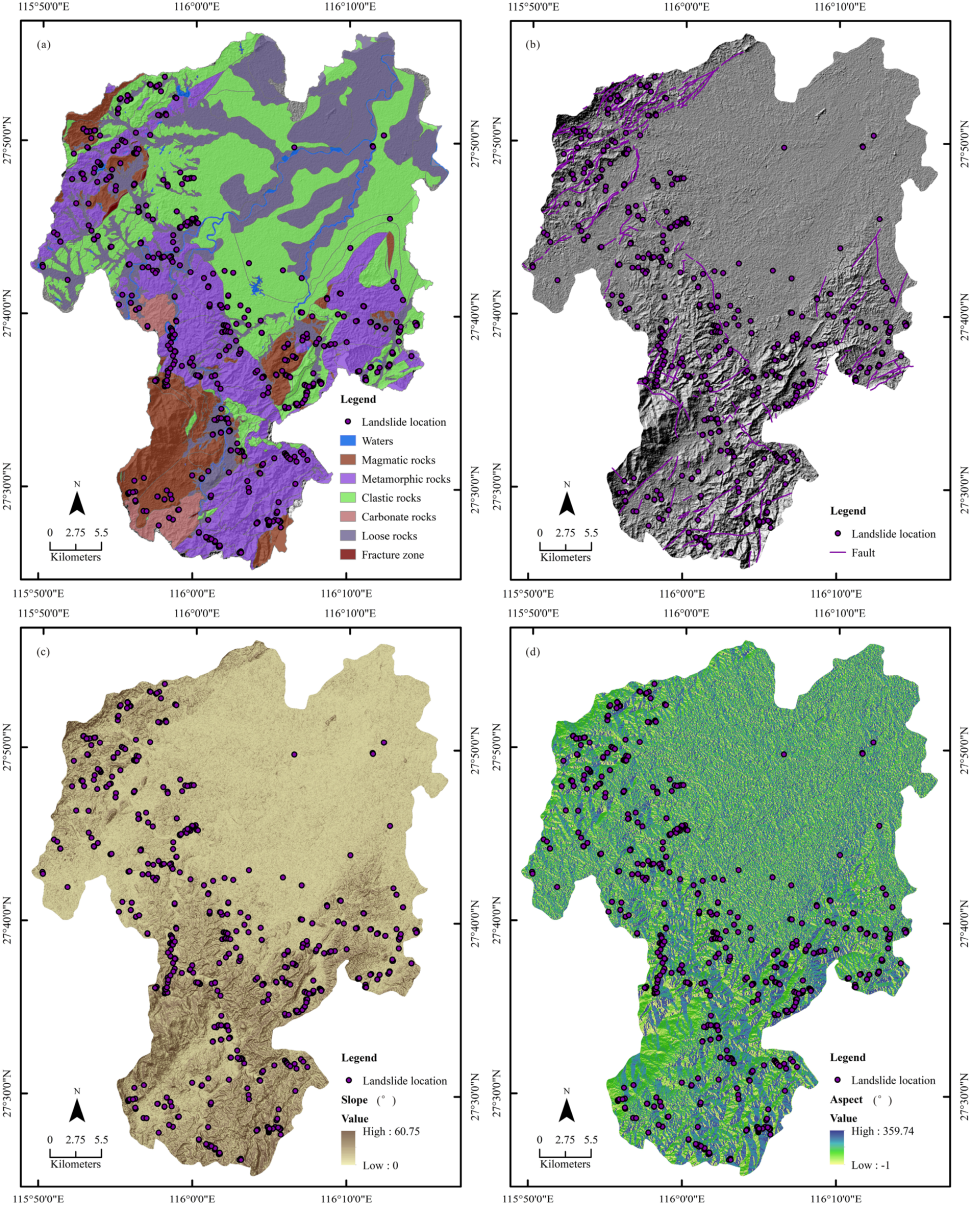
Geo-environmental factors: (**a**) lithology; (**b**) fault; (**c**) slope; (**d**) aspect. The maps were created using ArcGIS version 10.6 (https://www.esri.com/).

Geological factor layers such as lithology, geological boundary and faults were generated by vectorization, buffering, and rasterization from the 1/50,000 Geological Map (Fig. 4a,b). The soil data including soil types and texture were provided by the Bureau of Jiangxi Coal Geology.

**Figure 5:**
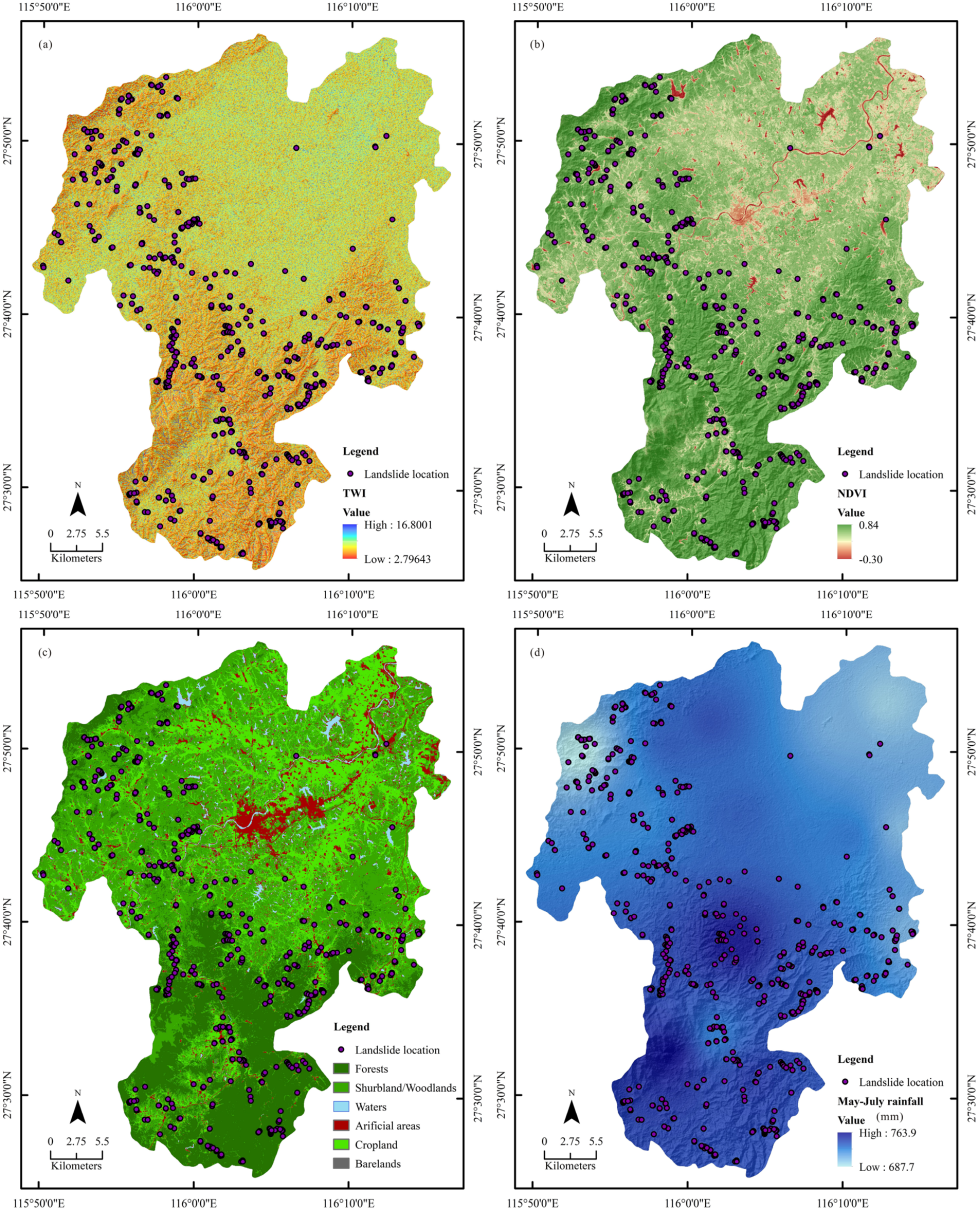
Geo-environmental factors: (**a**) TWI; (**b**) NDVI; (**c**) landuse; (**d**) May–July accumulated mean rainfall. The maps were created using ArcGIS version 10.6 (https://www.esri.com/).

Slope and aspect factor layers were extracted from the digital elevation model (DEM), ASTGTMV003 (30 m), which were obtained from NASA (www.earthdata.nasa.gov) (Fig. 4c,d). The topographic wetness index (TWI) was also calculated using DEM data (Fig. 5a), using Eq. (1)^20^:

1 $$ {\text{TWI}} = {\text{ln}}{\raise0.7ex\hbox{${A_{s} }$} \!\mathord{\left/ {\vphantom {{A_{s} } {{\text{tan}}\beta }}}\right.\kern-\nulldelimiterspace} \!\lower0.7ex\hbox{${{\text{tan}}\beta }$}} $$

where *A*_*S*_ is the upslope area of contribution per unit length of contour (m^2^/m), and *β* is the slope gradient.

The normalized difference vegetation index (NDVI) is a good representative of vegetation dynamics and can hence be considered as a controlling factor of landslide. For this reason, the multiyear autumn average NDVI was adopted to reduce the influence of uncertainty factors related to cloud cover and vegetation phenological change. Obtained from the USGS data server, Landsat 5 TM (30 m) and Landsat 8 OLI (30 m) images of the period 2007–2017 were used for this purpose. These Landsat images were acquired in late autumn, i.e., late October and early November, when crops are mostly harvested and only forests and woodlands are still green. After atmospheric correction using the COST model^45–47^, these Landsat images were employed for deriving the mean autumn NDVI (Fig. 5b), and Landsat 8 OLI images dated May 2017 and Sept 2019 were used for land cover mapping (Fig. 5c) using the approach developed by Wu et al.^29^.

Daily precipitation data from 2008 to 2017 were obtained from 14 meteorological stations in Chongren. Our previous studies revealed that the precipitation from May to July has a higher impact on the landslide occurrence than the combination of other months^27,28^. Thus, the May–July accumulated mean rainfall was generated by interpolation approach of the Inverse Distance Weighting (IDW) (Fig. 5d).

**Figure 6:**
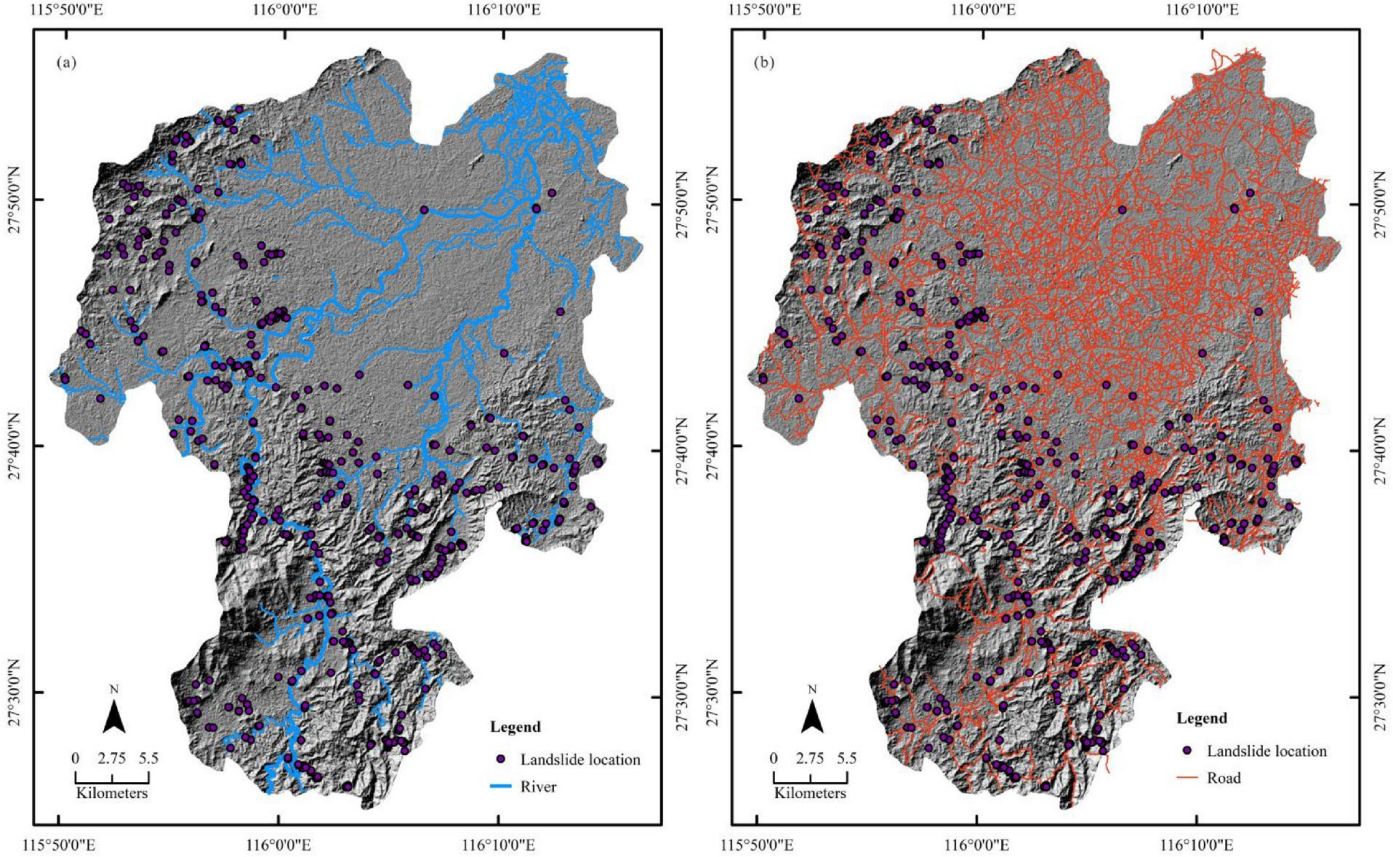
Geo-environmental factors: (**a**) rivers; (**b**) roads. The maps were created using ArcGIS version 10.6 (https://www.esri.com/).

Linear feature factors such as roads and rivers were vectorized from Google Earth (©Google) (Fig. 6a,b) and buffered into belts with intervals at 30, 60, 90, 120 and 150 m, respectively.

#### Optimal discretization of the continuous factors

The supervised discretization approach based on entropy was used to divide the continuous variables into intervals to realize optimal discretization. Using the entropy value to represent the purity of the dataset after partition is the basic idea of the approach. The smaller the entropy, the greater the data purity and the higher the availability of the discrete data obtained. The formula of entropy is presented as follows:

2 $$ E = \sum { - P_{i} \log_{2} P_{i} } $$

where *P*_*i*_ represents the probability of class *i* of sample appearing in the data interval. The results of division for continuous factors are shown in Table 1.

**Table 1:** The weight contrasts (C) of the geo-environmental factors.

Factor	Class	Area of intervals (km^2^)	Number of landslides	W^+^	W^-^	C
Lithology	Magmatic rocks	177.67	138	1.22	− 0.20	1.42
	Metamorphic rocks	363.12	199	0.87	− 0.26	1.13
	Clastic rocks	535.62	127	0.03	− 0.01	0.04
	Carbonatic rocks	38.56	12	0.31	− 0.01	0.31
	Quaternary sediments	380.69	106	0.20	− 0.04	0.23
Geological boundary (m)	0–30	109.88	43	1.15	− 0.05	1.20
	30–60	105.46	51	1.36	− 0.07	1.43
	60–90	96.85	34	1.04	− 0.04	1.08
	90–120	87.93	37	1.22	− 0.05	1.26
	> 120	4309.44	423	− 0.24	1.16	− 1.40
Fault (m)	0–60	50.96	47	2.02	− 0.07	2.09
	60–120	51.68	47	2.00	− 0.07	2.08
	120–180	51.55	33	1.65	− 0.05	1.70
	180–240	49.95	30	1.59	− 0.04	1.63
	240–300	47.86	22	1.32	− 0.03	1.35
	> 300	4518.63	409	− 0.30	1.67	− 1.98
Soil type	Yellow–red soil	39.19	9	0.01	0.00	0.01
	Paddy soil	411.39	124	0.28	− 0.06	0.34
	Red soil	15.32	0	0.00	0.00	0.00
	Neutral skeletal soil	1031.71	449	0.65	− 0.93	1.58
Clay (%)	0–15.58	434.99	− 0.55	0.26	− 0.11	0.37
	15.58–24.38	757.90	− 3.53	0.16	− 0.16	0.32
	24.38–26.26	30.51	1.32	− 2.88	0.02	− 2.90
	26.26–37.97	194.96	− 0.99	− 0.16	0.02	− 0.18
	37.97–37.98	98.73	− 0.55	− 4.21	0.06	− 4.27
Sand (%)	0–22.57	360.09	60	− 0.45	0.10	− 0.55
	22.57–25.06	98.73	1	− 3.47	0.06	− 3.53
	25.06–61.93	838.92	481	0.44	− 0.89	1.32
	61.93–61.94	219.35	46	− 0.90	0.09	− 0.99
Elevation (m)	1–67	24.95	1	− 1.74	0.01	− 1.74
	67–82	358.67	208	0.94	− 0.29	1.22
	82–347	163.95	54	0.37	− 0.03	0.40
	347–1218	900.69	313	0.42	− 0.33	0.76
Slope (°)	0–9.51	976.12	281	− 0.17	0.21	− 0.38
	9.51–60.75	644.04	307	0.21	− 0.17	0.38
	11.44–18.34	65.38	24	0.48	− 0.02	0.50
Aspect	Flat	38.36	1	− 1.55	0.01	− 1.56
	North	598.05	63	− 0.15	0.02	− 0.17
	Northeast	611.22	78	0.04	− 0.01	0.05
	East	560.31	74	0.07	− 0.01	0.08
	Southeast	613.75	70	− 0.07	0.01	− 0.08
	South	603.14	98	0.28	− 0.05	0.33
	Southwest	582.89	78	0.09	− 0.01	0.10
	West	555.59	77	0.12	− 0.02	0.14
	Northwest	620.55	49	− 0.44	0.05	− 0.49
TWI	2.80–5.21	315.60	154	0.76	− 0.17	0.94
	5.21–6.26	561.57	234	0.60	− 0.26	0.87
	6.26–7.46	373.10	123	0.37	− 0.08	0.45
	7.46–9.00	187.68	52	0.20	− 0.02	0.21
	9.00–16.80	79.20	25	0.33	− 0.01	0.34
NDVI	− 0.30 to 0.25	36.25	1	− 2.03	0.02	− 2.05
	0.25 to 0.55	349.08	332	0.92	− 0.53	1.45
	0.55 to 0.68	579.05	225	0.17	− 0.11	0.27
	0.68 to 0.75	420.29	30	− 1.97	0.26	− 2.24
	0.75 to 0.84	132.42	0	0	0	0
Land use	Forest	337.22	37	− 0.73	0.07	− 0.80
	Woodland	589.43	185	0.32	− 0.12	0.44
	Artificial area	117.12	187	1.95	− 0.34	2.29
	Cropland	366.13	139	0.51	− 0.12	0.63
	Shurb	67.02	27	0.57	− 0.02	0.59
	Bareland	6.44	12	2.10	− 0.02	2.12
May–July mean rainfall (mm)	687.7–693.14	11.46	23	2.12	− 0.05	2.17
	693.14–738.75	1008.95	277	− 0.23	0.30	− 0.53
	738.75–763.90	496.68	288	0.38	− 0.23	0.61
River (m)	0–30	55.45	18	0.97	− 0.02	0.99
	30–60	52.14	20	1.14	− 0.02	1.16
	60–90	48.94	30	1.61	− 0.04	1.65
	90–120	46.11	34	1.79	− 0.05	1.84
	> 120	4573.73	486	− 0.14	1.33	− 1.48
Road (m)	0–30	174.65	52	0.87	− 0.05	0.93
	30–60	158.86	32	0.48	− 0.02	0.50
	60–90	142.32	29	0.49	− 0.02	0.51
	90–120	126.23	40	0.93	− 0.04	0.98
	> 120	4105.85	435	− 0.16	0.69	− 0.85

#### WoE-based processing of geo-environmental factors

Originally developed for mineral potential mapping based on Bayesian probability by Bonham-Carter et al.^48^, WoE has been introduced into the prediction of landslide hazard in recent years and achieved a good result^15^. The weight values of the evidential variables (i.e., geo-environmental factors) are statistically calculated by the spatial relationship of landslide events with geo-environmental factors^7,49^.

The positive weight (W^+^) and negative weight (W^−^) are provided by the following equations:

3 $$ W^{ + } = \ln \frac{{P(B{|}D)}}{{P(B|\overline{D})}} $$

4 $$ W^{ - } = \ln \frac{{P(\overline{B}|D)}}{{P(\overline{B}|\overline{D})}} $$

where *W*^+^ and *W*^*−*^ are the weighted values of the occurrence and non-occurrence of the observed geo-environmental factor, respectively. *B* and $\overline{B}$ is occurrence and non-occurrence of the geo-environmental factor, respectively; *D* and $\overline{D}$ are the occurrence and non-occurrence of landslide events, respectively; *P* is the probability^7,49^.

The weight contrast (C) is a global measurement of the spatial interconnection between the landslide points and the geo-environmental factors, incorporating the effects of the W^+^ and W^−^. Calculation of C is shown as follows^48^:

5 $$ C = W^{ + } - W^{ - } $$

where if C is > 0, it indicates that the occurrence of landslide is positively correlated with the geo-environmental factor; and if C is < 0, it implies that the occurrence of landslide is negatively correlated with the geo-environmental factor. The weight of evidence values of all the geo-environmental factors are shown in Table 1.

Each interval of the divided continuous factors and each type of feature within the categorical factor were considered as a “subset”. The positive weight (W^+^) and negative weight (W^−^) of different intervals or subsets for the geo-environmental factors were calculated using Eqs. (3) and (4). Lithology, soil type, soil texture, distance to faults, distance to geological boundary, distance to rivers, distance to roads, elevation, slope, aspect, TWI, autumn mean NDVI, May–July accumulated mean rainfall and land use were transformed into raster layers with 30 m resolution as input variables (e.g., C values) for WoE-based hybrid modeling.

The calculation of WoE and C are implemented within Arc-WofE, an extension to ArcView 3.3 developed jointly by the USGS and the Geological Survey of Canada^50^.

### Machine learning modeling

Based on the WoE calculation, the following machine learning algorithms were applied for landslide susceptibility modeling, or rather, hybrid modeling. LR model was established within SPSS 19.0 software, meanwhile, SVM and RF modeling was implemented within EnMap-Box 2.11, a software package developed using Interactive Data Language (IDL)^51^.

#### LR modeling

1. Collinearity analysis
  Prior to the LR modeling, it is necessary to understand the collinearity among the independent variables, that is to say, to ascertain whether there exists linear correlation among the independent geo-environmental factors. This collinearity may lead to an instability of the LR model and affect the contribution of variables to the model^52^. Common indicators to evaluate the collinearity of geo-environmental factors are the variance inflation factor (VIF) and tolerances (TOL)^53^. The statistical model and LR require that there be no collinearity among the factors, that is, TOL > 0.1 and VIF < 10^27,54^.
2. LR modeling

LR is an algorithm that learns a model for binary classification^46,55^ whose kernel function is sigmoid (Eq. 6).

6 $$ p(x) = \frac{1}{{1 + e^{ - x} }} $$

The purpose of the conventional regression algorithms is to fit a polynomial function (Eq. 7) that minimizes the error between the prediction and the reality.

7 $$ f(x) = c_{0} + c_{1} x_{1} + \ldots + c_{n} x_{n} $$

where *x*_*i*_ (*i* = 1, 2, 3, … *n*) are independent features of the samples; *c*_*i*_ (*i* = 1, 2, 3, … *n*) are the coefficients of the features, and *c*_0_ is a constant. *f*(*x*) is transformed into a sigmoid function so that it has a good logistic judgment property and can directly express the probability in which the sample with the given features is classified into a certain class. *p*(*x*) = 1 is the probability of samples being assigned to category 1, then *p*(x)/(1 − *p*(x)) is defined as odds ratio (OR) to introduce the natural logarithm (Eq. 8).

8 $$ f(x) = {\text{ln}}(\frac{p(x)}{{1 - p(x)}}) $$

*p*(*x*) is expressed as following function (9):

9 $$ p(x) = \frac{1}{{1 + e^{{ - (c_{0} + c_{1} x_{1} + \ldots + c_{n} x_{n}^{{}} )}} }} $$

The training samples and their corresponding attributes of environmental factors were inputted into a statistic package SPSS 19.0 to calculate the coefficients of environmental factors. Then, in the GIS environment, the probability of landslide occurrence in the study area was calculated through formula (9).

#### SVM modeling

As a classical classification and regression algorithm, SVM has clear advantages in dealing with high-dimensional data with limited samples. SVM attempts to find or construct a set of hyperplanes through kernel functions to separate clusters that are usually not linearly separable in low-dimensional feature space, minimizing the empirical error and uncertainty to improve the generalization performance^56,57^. The kernel functions include Linear, Polynomial, Sigmoid and Radial Basis Functions (RBF), among which the RBF, similar to Gaussian distribution and thus termed also Gaussian function (Eq. 10), performed best^29,30^ and has been widely used in classification and regression as it has fewer parameters and stronger flexibility^34^. The RBF kernel was hence used to establish the SVM model in this study.

10 $$ k\left( {x_{i} ,x_{j} } \right) = exp\left( { - g\left\| {x_{i} - x_{j} } \right\|^{2} } \right) $$

where *x*_*i*_ and *x*_*j*_ are the input vectors, and *g* is the width parameter of the Gaussian kernel function *k*.

#### RF modeling

RF is a decision-trees-based classification and regression algorithm that outputs the final outcome by voting all the results of these trees^58^. The classification decision-maker used in the RF algorithm is the Classification and Regression Tree (CART)^59^. The training samples of the decision-trees are obtained by randomly replaceable sampling in the original TS. The remaining samples, called the out-of-bag (OOB) data, are used for establishing an unbiased estimate of error during generalization and estimating the importance of each factor. The metric of attribute of CART in branch processing is Gini Coefficient (Eq. 11).

11 $$ Gini = 1{ - }\sum\limits_{i = 1}^{2} {p_{i}^{2} } $$

where *p*_*i*_ represents the probability of which the observed sample falls in category *i*, so the probability of this sample being misclassified is (1 − *p*_*i*_).

In order to distinguish each predictor in the ensemble classifier, a specific number of variables are stochastically selected for generating the necessary nodes in the decision-tree. This construction method enables the RF to further improve the prediction performance through the increase of the difference among the individual classification trees and to avoid over-fitting. The number of variables at each node can be the square root of all features or logarithm (log) of all features or a user-defined value. In this study, the square root of all features, 4, was selected.

### Model performance assessment

The confusion matrix is often used for evaluation of the performance of the ML models. It mainly includes the following basic indicators: True Positive (TP) is the number of landslide samples correctly predicted by the model; False Negative (FN) is the number of landslide samples wrongly predicted as stable points by the model; False Positive (FP) is the number of stable samples mistakenly classified as landslide samples; True Negative (TN) is the number of stable samples correctly predicted by the model. The performance indicators of landslide hazard model, e.g., Precision, Recall, F-measure, Kappa Coefficient (KC), Overall Accuracy (OA) and AUC [area under the Receiver Operating Characteristic (ROC) curve], were calculated on the basis of confusion matrix^8,34^.

According to previous studies, the smaller the very high susceptible zone and the more landslide samples predicted, the higher the accuracy of the landslide risk map^60^. To assess the accuracy of the latter, the FR was also calculated, which is the ratio of the percentage of the cell number of landslides at each susceptibility level to the percentage of the cell number of each hazard level^61^. For a reliable landslide prediction model, the very high risk level shall possess the highest FR.

## Results

### Collinearity of the geo-environmental factors

As demonstrated in Table 2, the minimum TOL and maximum VIF values of the variables processed by WoE method were 0.878 and 1.139, respectively. The collinearity of WoE-based variables was significantly lower than that of the original variables, in which the minimum TOL and the maximum VIF are 0.215 and 4.642, respectively. Processing based on WoE can effectively reduce the collinearity among the factors. The collinearity among the geo-environmental factors selected for this research is low, and thus, they can be used for susceptibility modeling.

**Table 2:** Regression coefficients (β) and collinearity of the variables.

Factors	LR			WoE-LR		
	β	TOL	VIF	β	TOL	VIF
Lithology	− 0.585	0.917	1.090	0.929	0.829	1.207
Geological boundary	− 0.028	0.962	1.039	1.017	0.910	1.099
Fault	− 0.005	0.962	1.039	0.804	0.942	1.062
Slope	0.049	0.723	1.383	0.014	0.928	1.077
Aspect	0.001	0.985	1.015	1.784	0.977	1.024
Elevation	− 0.002	0.643	1.555	0.837	0.889	1.125
Land use	0.304	0.735	1.361	1.200	0.560	1.785
NDVI	− 5.636	0.743	1.345	0.784	0.549	1.822
May–July accumulated mean rainfall	0.023	0.862	1.160	1.689	0.812	1.232
River	0.017	0.984	1.017	− 0.844	0.962	1.039
Road	− 0.030	0.953	1.049	0.977	0.856	1.168
Sand	− 0.003	0.990	1.010	0.516	0.958	1.043
Clay	− 0.156	0.215	4.642	0.655	0.878	1.139
Soil type	0.865	0.974	1.027	− 1.104	0.971	1.030
TWI	− 0.136	0.960	1.041	1.119	0.977	1.023
Constant	− 8.685			− 0.033		
R^2^	0.707			0.886		

### Hybrid models

#### WoE-based LR models

Regression coefficient (β) and R^2^ of the WoE-based LR model is shown in Table 2. The single LR model was also established for comparison. The fitting degree of the WoE-based LR Model (R^2^ = 0.886) was better than that of the single model (R^2^ = 0.707). The WoE-based LR and single LR model were expressed using Eqs. (12) and (13). The probabilities of the landslide are calculated as follows:

12 $$ p(x) = \frac{1}{{1 + e^{{ - ({ - }8.685{ - }0.028x_{1} + \ldots { - }0.136x_{15}^{{}} )}} }} $$

13 $$ p(x) = \frac{1}{{1 + e^{{ - (1.119 + 0.929x_{1} + \ldots + 1.119x_{n}^{{}} )}} }} $$

where *x*_1_-lithology, *x*_2_*-*geological boundary, *x*_3_-fault, *x*_*4*_-slope, *x*_5_-aspect, *x*_6_-elevation, *x*_7_-land use, *x*_8_-NDVI, *x*_9_-May–July mean rainfall, *x*_10_-river, *x*_11_-road, *x*_12_-sand, *x*_13_-clay, *x*_14_-soil type and *x*_15_-TWI.

According to the modeled probability of each cell, the landslide risk zoning maps from WoE-based LR and the single LR model were created.

#### WoE-based SVM model

The width parameter *g* and the regularization parameter *c* of the optimal Gaussian kernel function were obtained by using the internally validated 2D grid search method, which were 1, 0.1 and 0.1, 100 in the WoE-based SVM and the single SVM model respectively. The *c* parameter indicates the penalty level for the error item^8^. The *c* value of the single SVM model was much higher than that of the WoE-based SVM model, implying that the penalty of the single SVM model for misclassification of the samples in the training process was bigger than that of the WoE-based SVM model, implying that the latter has stronger generalization capacity.

#### WoE-based RF model

The number of decision-trees (NT) has an important effect on the accuracy of RF model. The prediction performance of RF is poor when NT is small, and it becomes better when NT is larger. However, with the increase of NT, the complexity of RF model gradually increases, and the modeling time is also longer. Several experiments show that when NT was increased to 300, the prediction performance of RF was stable^28^. Based on this, the RF model for predicting landslide hazard was established with the NT of 300.

### Landslide susceptibility maps (LSM)

**Figure 7:**
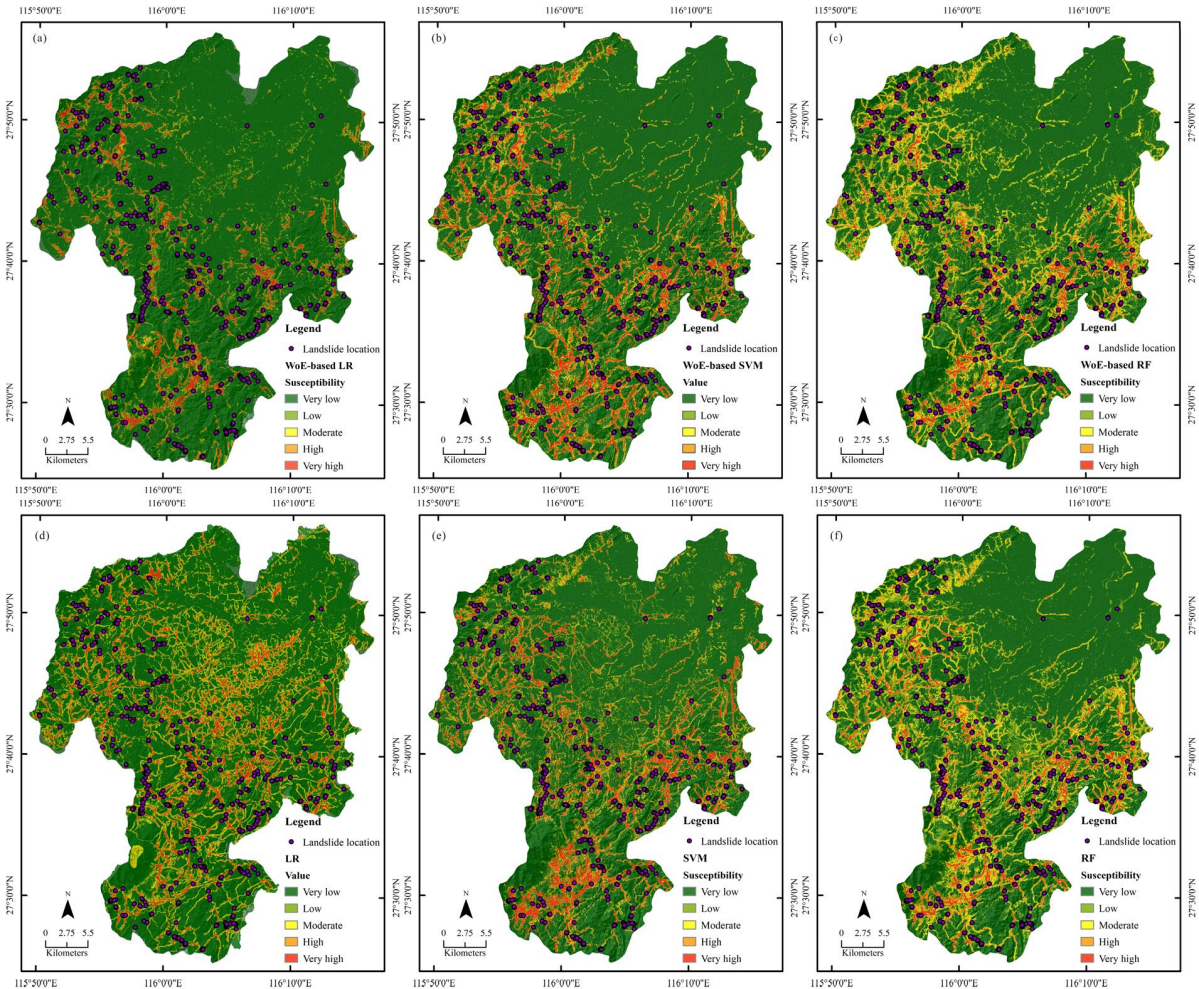
Landslide susceptibility zoning maps from different hybrid models: (**a**) WoE-based LR; (**b**) WoE-based SVM; (**c**) WoE-based RF; (**d**) single LR; (**e**) single SVM; (**f**) single RF model. The maps were created using ArcGIS version 10.6 (https://www.esri.com/).

The generated probability of landslide occurrence from the above hybrid models was reclassified into five levels: 0–0.2, 0.2–0.4, 0.4–0.6, 0.6–0.8 and 0.8–1, representing the five levels of landslide susceptibility, i.e., very low, low, moderate, high and very high, and the zoning maps are presented in Fig. 7. It is seen that most of the occurred landslides are distributed along the roads.

As revealed in Table 3, the very high susceptibility areas of the WoE-based LR and single LR, the WoE-based SVM and single SVM, the WoE-based RF and single RF were 88.80 km^2^, 110.78 km^2^, 137.47 km^2^, 110.78 km^2^, 77.87 km^2^, 79.13 km^2^, respectively, accounting for 5.94%, 7.30%, 9.06%, 8.71%, 5.93% and 6.43% of the studied territory, respectively. In all landslide susceptibility maps, FR values range from 0.01 to 14.05, and the very low risk level had also the very low FR and vice versa. With the increase of the susceptibility level, the area of the corresponding level decreases and the percentage of landslides increases, denoting the high prediction accuracy by all the coupled hybrid models. Our analysis also exhibits that the WoE-based RF modeling map grasps the highest FR but with the least surface area at very high risk level, indicating that this hybrid model performs better than others and may allow us to target accurately the zones for implementing landslide risk reduction and prevention measures.

**Table 3:** Landslide distribution with different susceptibility levels.

Model	Geohazard level	Area (km^2^)	Number of historical landslides	Proportion of landslides (%)	Proportion of levels (%)	FR
WoE-based LR	Very low	1244.87	116	19.73	83.25	0.24
	Low	73.24	44	7.48	4.90	1.53
	Moderate	45.58	46	7.82	3.05	2.57
	High	42.87	63	10.71	2.87	3.74
	Very high	88.80	319	54.25	5.94	9.14
Single LR	Very low	1017.92	54	9.18	67.03	0.14
	Low	181.03	51	8.67	11.92	0.73
	Moderate	112.91	49	8.33	7.44	1.12
	High	95.98	108	18.37	6.32	2.91
	Very high	110.78	326	55.44	7.30	7.60
WoE-based SVM	Very low	1101.38	15	2.55	72.59	0.04
	Low	123.77	27	4.59	8.16	0.56
	Moderate	79.05	24	4.08	5.21	0.78
	High	75.50	43	7.31	4.98	1.47
	Very high	137.47	479	81.46	9.06	8.99
Single SVM	Very low	1087.54	31	5.27	71.68	0.07
	Low	140.88	18	3.06	9.29	0.33
	Moderate	83.36	26	4.42	5.49	0.80
	High	73.30	50	8.50	4.83	1.76
	Very high	132.09	463	78.74	8.71	9.04
WoE-based RF	Very low	977.28	4	0.68	64.42	0.01
	Low	236.35	14	2.38	15.58	0.15
	Moderate	135.68	24	4.08	8.94	0.46
	High	89.97	100	17.01	5.93	2.87
	Very high	77.87	446	75.85	5.13	14.78
Single RF	Very low	935.89	6	1.02	61.69	0.02
	Low	262.60	23	3.91	17.31	0.23
	Moderate	142.03	29	4.93	9.36	0.53
	High	97.52	99	16.84	6.43	2.62
	Very high	79.13	431	73.30	5.22	14.05

### Comparison of the LSMs

As shown in Table 4, the statistic indicators based on the confusion matrix show that the OA and KC of the coupled hybrid models, i.e., WoE-based LR, WoE-based SVM and WoE-based RF, were 82.35%, 87.86%, 91.20% and 0.6470, 0.7573, 0.8199 respectively, and the OA and KC of the single models of LR, SVM and RF were 76.75%, 81.00%, 89.00% and 0.5350, 0.6210, 0.7800 respectively. It is evident that the coupled hybrid models are able to effectuate a prediction with higher accuracy than the single models, and the WoE-based RF model had the highest OA and KC, and hence performed best. In accordance with the FR calculated by the landslide risk map, the accuracy and reliability of the coupled models with WoE-based variables are improved with regard to the single prediction model.

**Table 4:** The statistic indicators based on the confusion matrix versus the validation set (VS).

Item	WoE-based LR	LR	WoE-based SVM	SVM	WoE-based RF	RF
Precision (%)	78.50	69.75	86.11	74.02	91.67	88.24
Recall (%)	88.70	93.79	90.29	86.78	91.24	90.00
F-measure	83.29	80.02	88.15	79.89	91.45	89.11
KC (%)	64.70	53.50	75.73	62.10	81.99	78.00
OA (%)	82.35	76.75	87.86	81.00	91.02	89.00

**Figure 8:**
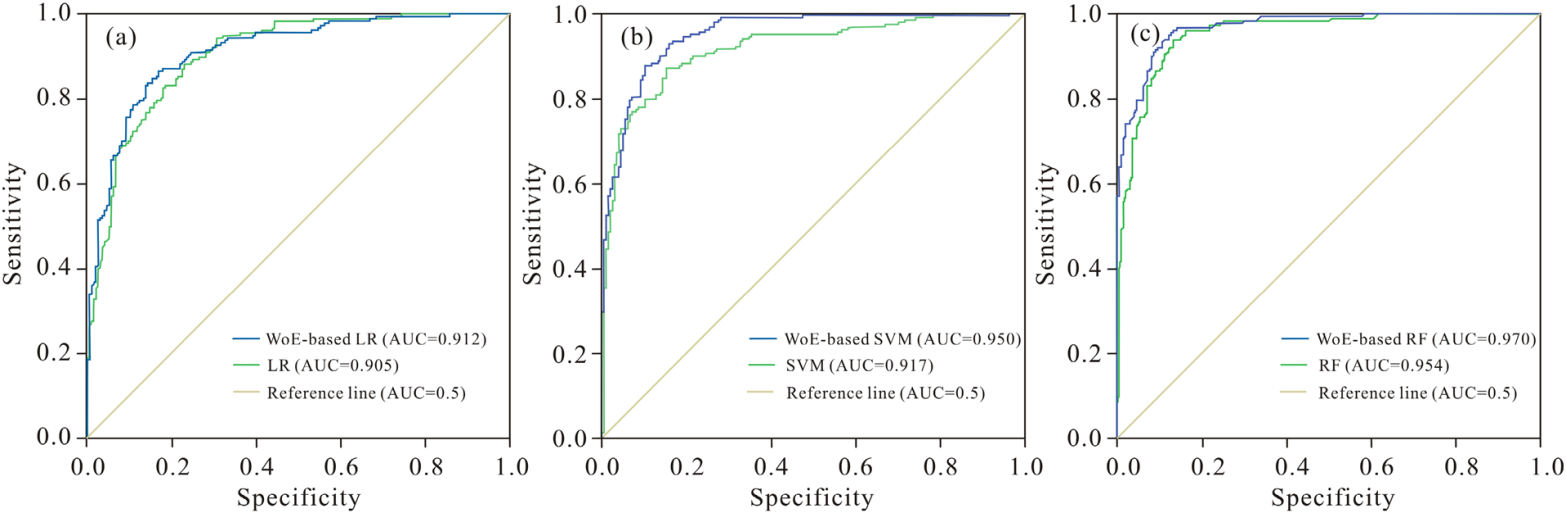
ROC curves with associated AUC values versus validation set (VS): (**a**) WoE-based LR and LR; (**b**) WoE-based SVM and SVM; (**c**) WoE-based RF and RF model.

The ROC curves and AUC of the coupled hybrid models in this study are shown in Fig. 8. It is seen that AUC of the WoE-based LR, WoE-based SVM and WoE-based RF are 0.912, 0.950 and 0.970 respectively, and that of the single models of LR, SVM and RF are 0.905, 0.917, 0.954, respectively.

## Discussion

### Advantages of the hybrid modeling

Based on the optimal discretization of the continuous factors, the WoE approach itself is able to provide the probability information of landslide in line with the a priori knowledge of the contribution of each geo-environmental factor to the historical landslides^15^. This should be favorable for the successive ML modeling of the landslide susceptibility. As a preprocessing approach, WoE has the following advantages: (1) the response degree of different subsets or intervals of these factors to landslide occurrence is quantitatively evaluated by the evidence weight; (2) the categorical variables are converted into numerical ones without subjective assignment; (3) the interference of outliers to the model is reduced by providing evidence weights to the geo-environmental factors. Hence, the WoE can simplify the ML processes and improve their prediction accuracy.

This research illustrates that WoE-based ML modeling performs better than single ML model and may lead to a reliable prediction, and the RF algorithm performs better than LR and SVM algorithms. The integration and random sampling characteristics make the RF model to have clear advantages over the others in the following aspects: (1) prediction less affected by the disturbance of data, (2) higher accuracy, and (3) more effective to prevent over-fitting thanks to using the Strong Law of Large Numbers for construction of the decision-trees. Some authors have specifically discussed the performance of ML models in predicting landslide hazard and showed that the RF algorithm may derive a higher prediction accuracy than other models, and is hence more suitable for landslide susceptibility mapping^11,14,18,27,28,62,63^. Our result is consistent with the conclusions of these authors.

### Comparison with other researches

As above mentioned, the reasonable processing, e.g., discrete processing of the continuous geo-environmental factors, together with WoE can improve the performance of ML models^10,21,38^. In this research, the OA and KC of all the coupled models are better than those of single models, which reflects the usefulness of such preprocessing prior to ML modeling.

The landslide susceptibility of the Chongren area had also been modeled by other authors. The one of Hong et al.^64^ shows that the index of entropy (IOE) model obtains a better accuracy than other binary models with an AUC value of 0.817. Two other studies conducted by Chen et al.^62,65^ show that RF can achieve satisfactory results among the ML algorithms with an AUC value of 0.851. Compared with the existing works, even those conducted in other areas with deep learning techniques, the accuracy of this study, with AUC values of 0.912–0.970, is greatly improved. This implies the effectiveness of the WoE-based hybrid ML modeling and entropy-based optimal discretization of the continuous factors. Thus, the methodology proposed in this study is considered effective and extendable to other subtropical areas for landslide hazard mapping.

## Conclusions

This paper presents an integrated study on landslide hazard mapping taking Chongren county as an example. Though the single known ML algorithm including deep learning and even the hybrid models have been applied by other researchers, the methodology proposed in this study, composed of an integrated procedure as mentioned above, does make an improved landslide risk prediction possible.

Our study reveals the effectiveness of the hybrid modeling for landslide risk mapping in which the WoE was applied for preprocessing the geo-environmental factors and ML algorithms for modeling. The coupled hybrid models, e.g., WoE-based LR, WoE-based SVM and WoE-based RF, have higher precision and better generalization ability than the single models for landslide hazard prediction. We also note that the decision-tree-based ensemble algorithm has achieved rather satisfactory results in comparison with others and that the WoE-based RF model offers a robust landslide prediction, and will be hence recommended for the similar landslide prediction elsewhere.

As we have noted, road construction is the most important geo-environmental factor provoking landslides and this confirms what we have observed in previous studies^26–28,36^. This requires our attention to the potential disaster that may be induced while planning future urbanization and road development.

Another innovation of this research is using the optimal discretization approach for numeric factors prior to the application of the WoE approach. After this, the landslide susceptibility prediction based on ML algorithms becomes more reliable. We believe that our research provides an operational methodology for predicting the hazard of landslide and collapse in the subtropical area, and may serve better for local authorities to accurately target the risk zones to implement disaster early warning and prevention measures.
